# *In Vitro* Germ Cell Differentiation from Cynomolgus Monkey Embryonic Stem Cells

**DOI:** 10.1371/journal.pone.0005338

**Published:** 2009-04-28

**Authors:** Kaori Yamauchi, Kouichi Hasegawa, Shinichiro Chuma, Norio Nakatsuji, Hirofumi Suemori

**Affiliations:** 1 Laboratory of Embryonic Stem Cell Research, Stem Cell Research Center, Institute for Frontier Medical Sciences, Kyoto University, Sakyo-ku, Kyoto, Japan; 2 Department of Development and Differentiation, Institute for Frontier Medical Sciences, Kyoto University, Sakyo-ku, Kyoto, Japan; 3 Institute for Integrated Cell-Material Sciences (iCeMS), Kyoto University, Sakyo-ku, Kyoto, Japan; Institut de la Vision, France

## Abstract

**Background:**

Mouse embryonic stem (ES) cells can differentiate into female and male germ cells *in vitro*. Primate ES cells can also differentiate into immature germ cells *in vitro*. However, little is known about the differentiation markers and culture conditions for *in vitro* germ cell differentiation from ES cells in primates. Monkey ES cells are thus considered to be a useful model to study primate gametogenesis *in vitro*. Therefore, in order to obtain further information on germ cell differentiation from primate ES cells, this study examined the ability of cynomolgus monkey ES cells to differentiate into germ cells *in vitro*.

**Methods and Findings:**

To explore the differentiation markers for detecting germ cells differentiated from ES cells, the expression of various germ cell marker genes was examined in tissues and ES cells of the cynomolgus monkey (*Macaca fascicularis*). *VASA* is a valuable gene for the detection of germ cells differentiated from ES cells. An increase of *VASA* expression was observed when differentiation was induced in ES cells via embryoid body (EB) formation. In addition, the expression of other germ cell markers, such as *NANOS* and *PIWIL1* genes, was also up-regulated as the EB differentiation progressed. Immunocytochemistry identified the cells expressing stage-specific embryonic antigen (SSEA) 1, OCT-4, and VASA proteins in the EBs. These cells were detected in the peripheral region of the EBs as specific cell populations, such as SSEA1-positive, OCT-4-positive cells, OCT-4-positive, VASA-positive cells, and OCT-4-negative, VASA-positive cells. Thereafter, the effect of mouse gonadal cell-conditioned medium and growth factors on germ cell differentiation from monkey ES cells was examined, and this revealed that the addition of BMP4 to differentiating ES cells increased the expression of *SCP1*, a meiotic marker gene.

**Conclusion:**

*VASA* is a valuable gene for the detection of germ cells differentiated from ES cells in monkeys, and the identification and characterization of germ cells derived from ES cells are possible by using reported germ cell markers *in vivo*, including SSEA1, OCT-4, and VASA, *in vitro* as well as *in vivo*. These findings are thus considered to help elucidate the germ cell developmental process in primates.

## Introduction

Embryonic stem (ES) cells are pluripotent and can be used to study germ cell development [1]–[5]. Recent studies have demonstrated that mouse ES cells can differentiate into female and male germ cells *in vitro*, thus producing ovarian follicle-like structures [1], [4] and testicular germ cells 2, 3. However, it remains unclear whether primate ES cells including those from humans can undergo gametogenesis through meiosis *in vitro*, although immature germ cell differentiation from primate ES cells has been reported [5], [6], [7]. To obtain further information on germ cell differentiation from primate ES cells, this study examined the ability of cynomolgus monkey (*Macaca fascicularis*) ES cells to differentiate into germ cells *in vitro*.

Suitable germ cell markers are necessary to distinguish between germ cells and ES cells. *VASA* is specifically expressed in developing germ cells from the primordial to the postmeiotic stage in diverse organisms from *Drosophila* to humans [8]–[11], thereby making it a useful marker for ES cell-derived germ cells in mice and humans [2], [5]. Therefore, *VASA* is a potential marker for ES cell-derived germ cells in monkeys. With regard to other germ cell marker genes, in mice, *developmental pluripotency-associated protein 3 (DPPA3*, also known as *stella/PGC7)*, *interferon-induced transmembrane protein 3 (IFITM3*, also known as *fragilis)*, and *deleted in azoospermia-like (DAZL)* have been investigated as potential markers for ES cell-derived germ cells [3]. In humans, *stella-related (STELLAR), growth and differentiation factor 3 (GDF3)*, *NANOS*, *c-KIT*, and *DAZL,* have been examined as well [5], [12]. However, these germ cell marker genes are not appropriate for detecting germ cell differentiation from mouse and human ES cells because these genes are expressed in both ES cells and germ cells. Therefore, it is necessary to determine the expression patterns of *VASA* and other germ cell marker genes in monkeys, but only limited information is currently available [7], [13].

Several protocols for inducing germ cell differentiation from ES cells have been reported. In mice, germ cells have been generated from ES cells using monolayer culture [1], the formation of embryoid bodies (EBs) [2], [3], co-aggregation with BMP4-producing cells [2], and the use of mouse testicular cell-conditioned medium [4]. In humans, germ cell differentiation from ES cells via spontaneous EB formation, and EB formation with recombinant human bone morphogenetic proteins (BMPs) has been reported [5], [6]. In monkeys, methods for inducing germ cell differentiation from ES cells have not been reported except spontaneous germ cell differentiation by EB formation [7]. Therefore, it is very important to develop a suitable protocol to induce *in vitro* germ cell differentiation from monkey ES cells before non-human primate ES cells can be used as a model for *in vitro* differentiated germ cells.

The current study examined the expression of germ cell marker genes in tissues and ES cells of the cynomolgus monkey, and the expression of several germ cell marker genes including *VASA* was confirmed. The up-regulation of *VASA* expression was observed in ES cells differentiated via spontaneous EB formation. The expression of other germ cell marker genes, such as *NANOS1*, *NANOS2*, *NANOS3* and *PIWIL1*, increased in the EBs as well. SSEA1-, OCT-4-, and VASA-positive cells were detected in the EBs, indicating that monkey ES cells have the ability to differentiate into a germ cell lineage *in vitro*. In addition, the effects of mouse testicular [4] and ovarian cell-conditioned media, and also BMP4, RA, and SCF, on germ cell differentiation in monkeys were also investigated.

## Materials and Methods

### Animals and tissue collection

Tissues from 3- and 5-year-old cynomolgus monkey were purchased from Japan SLC (Hamamatsu, Japan) and Keari Company (Osaka, Japan), respectively. The tissues were dissected into small pieces, fixed with 4% paraformaldehyde (PFA) in phosphate-buffered saline (PBS), and washed with PBS. The samples were then soaked in 30% sucrose at 4°C, immersed in a 1∶1 mixture of 30% sucrose and OCT compound (OCT; Tissue-Tek 4583; Sakura Finetechnology, Tokyo, Japan) at room temperature, and embedded in OCT. Some pieces from the 5-year-old monkey testis were fixed in Bouin's fixative and embedded in paraffin. For the histological analysis, the samples were sectioned into 5-µm thickness and stained with hematoxylin and eosin.

### Cell culture and the formation of embryoid bodies

A cynomolgus monkey ES cell line, CMK6, was cultured as previously described [14]. To produce EBs, ES cell colonies were removed from feeder cells using a detaching solution, which consisted of 0.25% trypsin supplemented with 1 mg/ml collagenase IV, 1 mM CaCl_2_, and 20% knockout serum replacement (KSR; Invitrogen, Carlsbad, CA). The colonies were cultured in suspension in Petri dishes with ES cell medium [14], and the culture medium was changed every 3 days. The resulting EBs were collected on days 3, 7, 14, 21, and 28. In the case of EB formation using mouse testicular or ovarian cell-conditioned medium (see below), the EBs were cultured in ES medium for 24 hr, and then in a conditioned medium. EBs were cultured in 6-well ultra-low attachment plates (Corning, Lowell, MA), and the culture medium was changed every 3 days. After 7, 14, 21, and 28 days, the EBs were collected for RT-PCR analysis. To investigate EB formation in the presence of growth factors, 100 ng/ml recombinant human BMP4 (R&D Systems, Minneapolis, MN), 1 µM all *trans*-retinoic acid (RA; Sigma-Aldrich, St. Louis, MO), and 100 ng/ml recombinant human stem cell factor (SCF; R&D Systems), the EBs were cultured in Dulbecco's Modified Eagle's Medium (DMEM; Sigma) supplemented with 20% fetal bovine serum (FBS; HyClone, Boston, MA), 1 mM minimal essential medium (MEM) nonessential amino acids, 2 mM L-glutamine, 0.1 mM 2-mercaptoethanol, and penicillin–streptomycin. Growth factors were added after the initial incubation for 24 hr.

### RT-PCR and a quantitative RT-PCR analysis

Total RNA from monkey tissues was extracted using Sepasol RNAI (Nacalai Tesque, Kyoto, Japan) according to the manufacturer's instructions. Total RNA from ES cells or EBs was isolated using the RNeasy Mini Kit (Qiagen, Hilden, Germany). RT-PCR was performed using Superscript II Reverse Transcriptase (Invitrogen) and Ex-Taq Polymerase (Takara Biotechnology, Tokyo, Japan). To amplify the germ cell marker genes, primers were designed based on the human sequences as shown in Table 1. Quantitative RT-PCR was performed using SYBR Green RT-PCR reagents (Applied Biosystems, Foster City, CA) and an ABI PRISM 7500 Sequence Detection System (Applied Biosystems) according to the manufacturer's instructions. All experiments were performed at least three times for each sample. The relative amounts of *VASA* were normalized against *GAPDH* using the comparative threshold cycle (C_T_) method [15].

**Table 1:** List of RT-PCR and quantitative RT-PCR primers

gene	Forward primer (5′- to -3′)	Reverse primer (5′- to -3′)	Annealing temperature (°C)
PRDM1	CGTGTCAGAACGGGATGAAC	AAACGACCCGAGGGTAGAAG	58
PRDM14	CCTGCACCATGCGATTTCAGG	TGCATGAGCAGCCATCATCCTC	58
PRMT5	CAAGTGTCCAGAGCCTTGGAAG	AGTGCGTAGCTTCAAATCCAGC	58
DPPA3	TCTGTAGGAGCAGCAGTCCT	TCTAGCATTCTCAGAAGGAT	60
IFITM3	CCCAACTATGAGATGCTCAA	CCAGATGTTCAGGCACTTGG	56
GDF3	AGACTTATGCTACGTAAAGGAGCT	CTTTGATGGCAGACAGGTTAAAGTA	56
c-KIT	AAGGACTTGAGGTTTATTCCT	CTGACGTTCATAATTGAAGTC	56
CXCR4	AGGGCCTGAGTGCTCCAGTAG	ATAGTCCCCTGAGCCCATTTC	58
NANOS1	GCTAGGTCTGCGCACCATCT	GTGCCTCCATTCATAAAATG	58
NANOS2	AACTTCTGCAAGCACAACGG	GGCTCCAGTCACCAGCAG	58
NANOS3	TCCCGGGCCATCTACCAG	GCTGACTGGATGCCCAGC	58
DAZL	ATGTTAGGATGGATGAGACTGAGATTA	CCATGGAAATTTATCTGTGATTCTACT	56
VASA	ATTCTTGGAAGTCAGAAGCAGAAG	CTGGTTGACCAATTCTCGAGT	56
PIWIL1	GAAGCAGCCTGTCTTGGTCAGC	GAATCAAAGCTCAAACCCCAGTCTC	60
PIWIL2	CAGGCAGAGGCCATGTATTT	AACATGCCGACC TCATGCT	56
MLH1	GAGACAGTGGTGAACCGCAT	CTTGATTGCCAGCACATGGT	56
SCP1	GACAAGGAAAAGCAGGTATC	TTGCTGTTCTGTTCTCAATA	56
SCP3	TGGAAAACACAACAAGATCA	GCTATCTCTTGCTGCTGAGT	56
OCT-4	GAGAACAATGAGAACCTTCAGGAGA	TTCTGGCGCCGGTTACAGAACCA	60
TEKT1	ACGCCGTGAGGATTGAGCCA	CACGACACAGCTCCACGTTC	60
GAPDH	GGATTTGGCCGTATTGG	TCATGGATGACCTTGGC	56
VASA^a^	ATTCTTGGAAGTCAGAAGCAGAAG	TCTGATAATGTGCAAAGATGGAGT	62
GAPDH^a^	GAAGGTGAAGGTCGGAGTC	CATTGATGGCAACAATATCC	62

a quantitative RT-PCR primers

### Immunostaining

For immunofluorescence staining, OCT-embedded 3- and 5-year-old testes were sectioned at 7-µm thickness. The monkey ES cells were fixed with 4% PFA in PBS for 20 min. The EBs were fixed with 4% PFA in PBS for 2 hr, soaked in 15% sucrose for 1 hr, embedded in OCT, and then sectioned at 7-µm thickness. The primary antibodies were goat anti-human VASA polyclonal antibodies (1∶500; R&D Systems), mouse anti-human DAZL monoclonal antibody (1∶200; AbD Serotec, Oxford, UK), rabbit anti-human SCP1 polyclonal antibodies (1∶2000; Novus Biologicals, Littleton, CO), mouse anti-OCT-4 monoclonal antibody (1∶500; C-10; Santa Cruz Biotechnology, Santa Cruz, CA), or mouse anti-stage-specific embryonic antigen (SSEA) 1 monoclonal antibody (1∶300; MC-480; Developmental Studies Hybridoma Bank, Iowa, IA). For secondary antibodies, the sections were incubated with Alexa Fluor 546-conjugated donkey anti-goat IgG antibody (Molecular Probes, Eugene, OR), Alexa Fluor 488-conjugated donkey anti-rabbit IgG antibody (Molecular Probes) or Alexa Fluor 488-conjugated donkey anti-mouse IgG antibody (Molecular Probes), Alexa Fluor 546-conjugated goat anti-mouse IgG_2b_, or Alexa Fluor 488-conjugated goat anti-mouse IgM, and then were counterstained with 1 µg/ml Hoechst 33258 for nuclear staining.

### Conditioned medium

Testicular cells were isolated from 1-day-old F_2_ male mice produced by interbreeding C57BL6×CBA F_1_ mice. The testicular cell-conditioned medium was collected as previously described [4]. To prepare ovarian cells, ovarian tissues were isolated from 1-day-old F_2_ female mice produced by interbreeding C57BL6×CBA F_1_ mice. The cells were dissociated with 0.25% trypsin and 1 mM EDTA in PBS (0.25% trypsin–EDTA) for 10 to 15 min and suspended at a concentration of 100,000 cells/ml in DMEM (Sigma) supplemented with 10% FBS, 1 mM MEM nonessential amino acids, and penicillin–streptomycin. The cells were then seeded into 12-well culture plates (1 ml/well; Falcon, Franklin Lakes, NJ). Upon reaching confluence, the ovarian cells were dissociated with 0.25% trypsin–EDTA and transferred into new plates at a dilution of 1∶2–3. The ovarian cell-conditioned medium was then collected every 3 days for 2 weeks.

## Results

### Expression of germ cell markers in the tissues from the cynomolgus monkey

**Figure 1:**
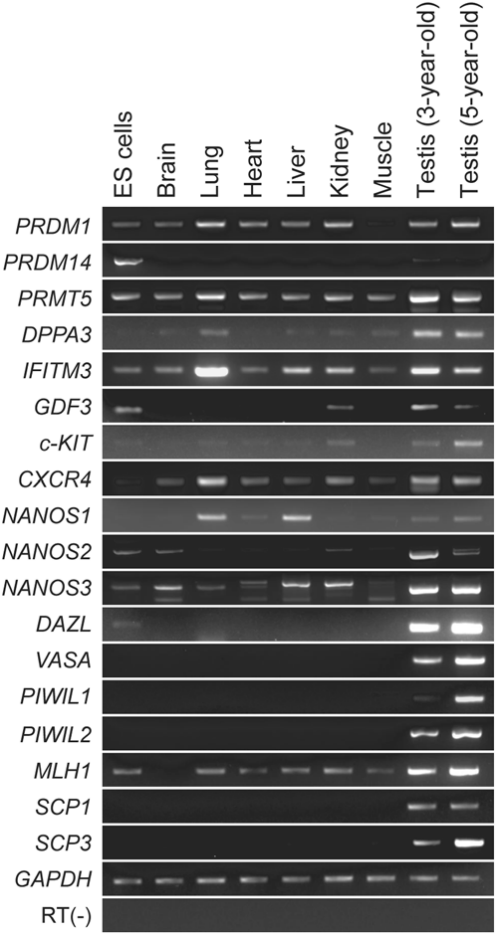
The expression of germ cell marker genes in tissues of the cynomolgus monkey. The expression of germ cell marker genes in monkey ES cells and tissues was examined using an RT-PCR analysis. *GAPDH* was used as an internal control.

To examine the expression patterns of germ cell markers in tissues of the cynomolgus monkey, we first performed RT-PCR analysis for germ cell marker genes reported in mice and humans, including *PR domain containing 1, with ZNF domain (PRDM1*, also known as *BLIMP1*) [16], *PR domain containing 14 (PRDM14)* [17], *protein arginine methyltransferase 5* (*PRMT5)* [18], *DPPA3* [19], [20], [21], *IFITM3* [20], [22], *GDF3* [23], [24], *c-KIT* [25], [26], *chemokine (C-X-C motif) receptor 4 (CXCR4)* [27], [28], *NANOS1-3* [29], [30], [31], *DAZL* [32]-[35], *VASA, PIWI family* genes (*PIWIL1* and *PIWIL2*, known as *HIWI* and *HILI* in humans, respectively) [36], [37], [38], *Mut-L Homologue-1* (*MLH1*) [39], [40], *synaptonemal complex protein 1* (*SCP1*) [41]–[44], and *SCP3* [42], [45], [46] (Figure 1). *PRDM1, PRMT5, DPPA3, IFITM3, GDF3,* and *c-KIT* expression was detected in various monkey tissues, including the testes and ES cells. Therefore, these early germ cell markers are not sufficiently specific to detect germ cells differentiated from ES cells. CXCR4, which is a chemokine receptor, is expressed in migratory and post-migratory germ cells *in vivo* in mice [27], and is also a marker for germ cells derived from human ES cells [28], thus suggesting that *CXCR4* is a candidate marker gene for detecting ES cells-derived germ cell in monkeys. However, the expression of *CXCR4* was observed in all monkey tissues including testes, thus indicating that this gene is also not sufficiently specific to distinguish germ cells from other differentiated cells in EBs. No expression of *PRDM14* was detected in somatic tissues examined. Although the *NANOS* genes did not show specific expression in testis, *NANOS2* and *3* were expressed predominantly in the testis. The expression of *DAZL*, *VASA,* and the *PIWI family* genes was detected in the testis, but not in the somatic tissues examined. Taken together, several marker genes, such as *PRDM14*, *DAZL*, *VASA*, and *PIWI family* genes could thus be candidates to detect germ cells derived from ES cells. With regard to the meiotic germ cell markers, *SCP1* and *SCP3* expression was also detected in the testes, whereas *MLH1* was expressed in various tissues and ES cells. Therefore, *SCP1* and *SCP3* were employed as markers to detect the meiotic stage in ES cell-derived germ cells.

**Figure 2:**
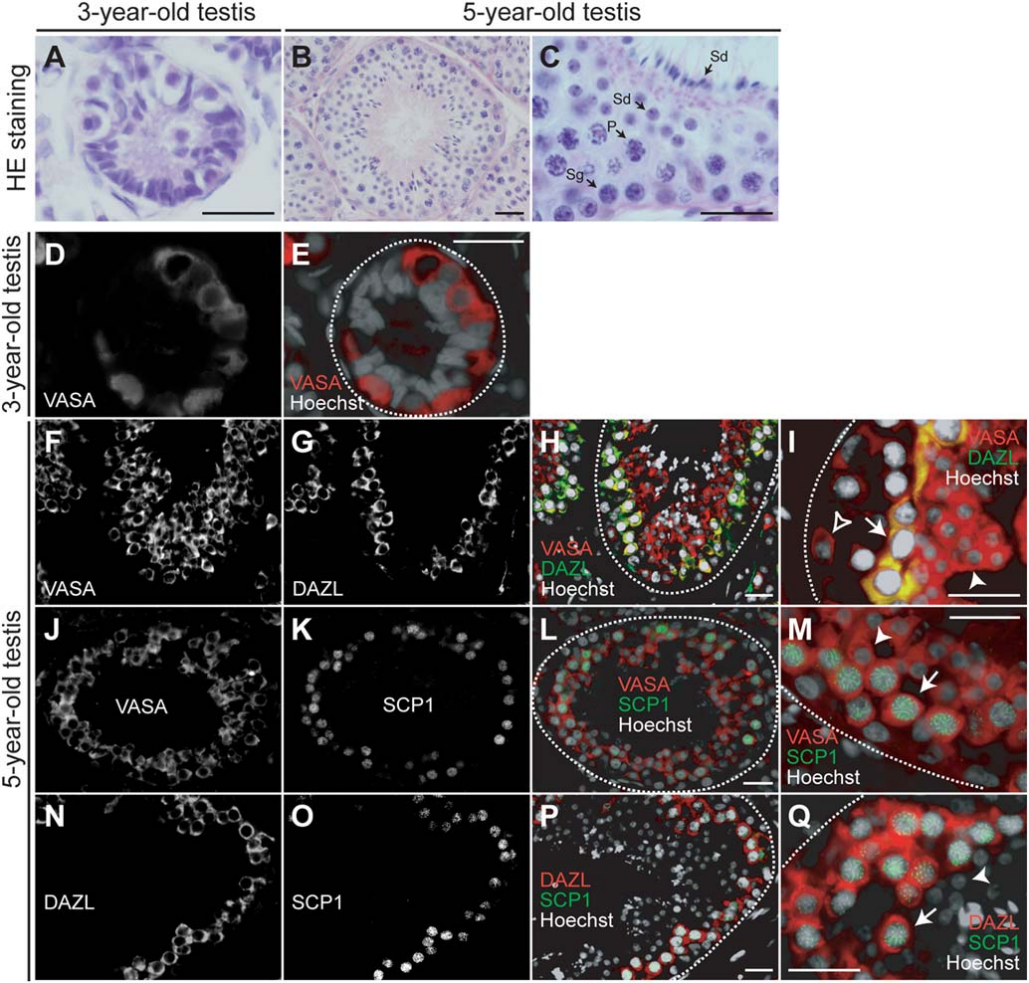
Morphological features of cynomolgus monkey testes, and expression of VASA protein. (A–C) Sections of testes of 3-year-old (A) and 5-year-old monkeys (B,C) were stained with hematoxylin and eosin. Sg, spermatogonium; P, pachytene spermatocyte; Sd, spermatid. (D–Q) Expression of VASA, DAZL, and SCP1 proteins during spermatogenesis in cynomolgus monkeys. Sections of 3-year-old (D,E) and 5-year old testes (F–Q) were examined by immunostaining. The nuclei were stained with Hoechst 33258. Merged images were also shown (E; VASA, red; Hoechst, white), (H, I; VASA, red; DAZL, green; Hoechst, white), (L,M; VASA, red; SCP1, green; Hoechst, white), (P,Q; DAZL, red; SCP1, green; Hoechst, white). VASA expression was observed in spermatogonia in the 3-year-old testis (E, red), and in spermatogonia (open arrowhead), spermatocytes (arrow), and early spermatids (arrowhead) in the 5-year-old testis (I, M, red). The expression of VASA, DAZL, and SCP1 proteins was all detected in spermatocytes (arrow; I, M, Q). The dotted lines indicate the basement membranes of the seminiferous tubules. The scale bar is 25 µm.

Next, 3- and 5-year-old monkey testes were analyzed histologically. In the 3-year-old, sexually immature monkey testis, spermatogonia were detected along the basement membranes of the seminiferous tubules (Figure 2A). In contrast, germ cells differentiated from spermatogonia to spermatids were observed in the seminiferous tubules of the 5-year-old testis (Figure 2B and 2C). VASA expression was examined in these monkey testes by immunohistological staining. VASA protein was detected in the cytoplasm of spermatogonia in the 3-year-old testis (Figure 2D and 2E) and in spermatogonia, spermatocytes, and early spermatids of the 5-year-old testis (Figure 2F–2M), however it was not observed in the somatic cells of either the immature or mature testis. The level of VASA expression in spermatogonia was relatively weak in comparison to that in spermatocytes and early spermatids (Figure 2I).

The expression patterns of VASA, DAZL and SCP1 were also compared. DAZL expression has been reported in gonocytes from fetal gonads and throughout gametogenesis in humans [47], [48]. The cytoplasmic co-localization of VASA and DAZL were observed in spermatocytes, and barely detectable in spermatogonia and spermatids in monkey testis (Figure 2F–I). SCP1 is expressed in primary spermatocytes as a component of the synaptonemal complex in mice and humans [43], [44]. VASA and SCP1 were expressed specifically in spermatocytes, but only VASA was expressed in spermatogonia and early spermatids (Figure 2J–2M). These results are consistent with previous reports on mice [49], [50] and humans [11], [51]. These results indicated that VASA was a useful marker for detecting germ cells, including meiotic cells, in cynomolgus monkeys, because VASA was expressed in the cells during the early to late stages of spermatogenesis.

### Germ cell differentiation from the monkey ES cells by EB formation

**Figure 3:**
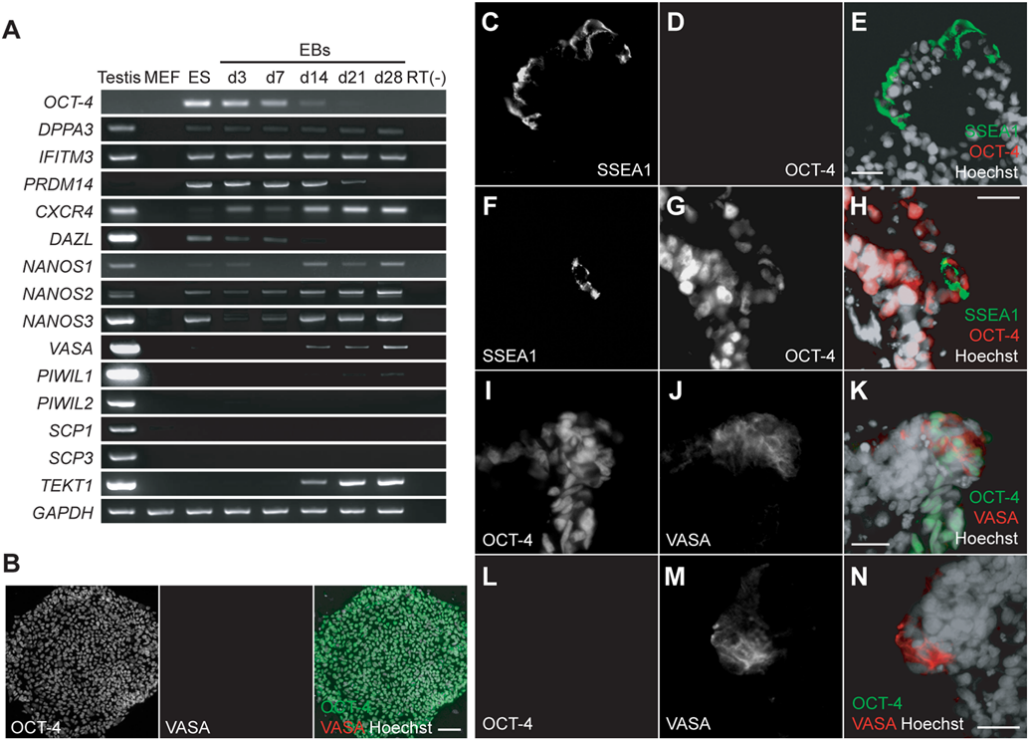
The expression of germ cell marker genes in ES cells of cynomolgus monkey during EB formation. (A) The expression patterns of germ cell marker genes in monkey testis (5 years old), mouse embryonic fibroblast (MEF), monkey ES cells (ES), and developing EBs (days 3, 7, 14, 21, and 28) were examined using an RT-PCR analysis. *GAPDH* was used as an internal control. (B–N) An immunocytochemical analysis of ES cells and day 14 EBs. ES cells were doubly immunostained with anti-OCT-4 and anti-VASA antibodies (B). Left column, OCT-4; middle column, VASA; right column, OCT-4 (green) and VASA (red) merged with Hoechst 33258 (white) staining. The sections of day 14 EBs were doubly immunostained with anti-SSEA1 and anti-OCT-4 (C–H) antibodies, or anti-OCT-4 and anti-VASA (I–N) antibodies. The expression of each protein is shown in left and middle columns. Merged images with Hoechst 33258 (white) staining are also shown in right column (E, H; OCT-4, red; SSEA1, green; K, N; OCT-4, green; VASA, red). The scale bars are 100 µm (B) and 25 µm (C–N), respectively.

To investigate the differentiation of monkey ES cells into germ cells *in vitro*, we produced EBs and examined the expression of germ cell markers using RT-PCR (Figure 3A). The expression of *OCT-4* decreased significantly in EBs by day 14, thus indicating that the differentiation of the monkey ES cells was induced by EB formation. The expression of *DPPA3* and *IFITM3* was observed in EBs from all stages examined. The *CXCR4* expression increased as EB differentiation progressed. The expression of *NANOS2* and *3* decreased at day 3, but thereafter increased dramatically from day 7 in the EBs, though both genes were expressed in ES cells. A similar increase of *NANOS1* expression was observed in the EBs after day 14. *VASA* expression was almost undetectable in ES cells and increased significantly in the EBs after day 14, thus suggesting that differentiation into the germ cell lineage occurs in the EBs. The expression of the *PIWI family* genes, *PIWIL1* and *PIWIL2*, was further examined in the EBs. A slight increase in *PIWIL1* expression was also detected in the EBs after day 21, whereas no *PIWIL2* expression was detected during EB differentiation. This observation suggests that *PIWIL1* could be a marker gene for the detection of ES cell-derived germ cells. *PRDM14* and *DAZL* were expressed in ES cells, but this expression in the EBs decreased by day 21 and 14, respectively. PRDM14 is required for the development of mouse germ cells [17], and also known as a pluripotent marker for mouse and human ES cells [17], [52]. The transient expression of PRDM14 in the inner cell mass of E3.5 embryo disappeared by E5.5 and the re-expression of which was continued in primordial germ cells until ∼E13.5–14.5 in mice [17]. Although the kinetics of *PRDM14* gene expression during EB development appeared to be similar to those during mouse embryogenesis, the expression pattern of the gene *in vitro* did not completely mimic the same pattern *in vivo*. Regarding *DAZL* expression, it has been reported in gonocytes from fetal gonads and throughout gametogenesis in humans [47], [48]. The expression pattern of *DAZL in vitro* appeared different from those *in vivo*. To examine whether EB-derived *VASA*-positive cells undergo meiosis, *SCP1* and *SCP3* expression was analyzed. *SCP1* and *SCP3* expression was not detected in EBs from any of the ages examined. In addition, the expression of *TEKT1*, a postmeiotic male germ cell marker [53], was examined during EB development. Unexpectedly, *TEKT1* expression was up-regulated in EBs from day 14 onward. In contrast, *GDF9*, which is an oocyte-specific early folliculogenesis marker [54], was not detected using RT-PCR (data not shown).

To further examine the differentiation of ES cells into a germ cell lineage, EBs were stained with anti-VASA antibodies. Although the VASA expression was not detectable in ES cells (Figure 3B), clusters of VASA-positive cells were found in EBs from day 14 (Figure 3J and 3M). To examine the proportion of VASA-positive cells in the EBs, sections of EBs (300 sections) were randomly selected and stained for VASA protein. The number of VASA-positive cells was counted and compared to the total number of cells in each section. The mean proportion of VASA-positive cells was 1.8±0.6% (n = 7) in the sections which contained VASA-positive cells. The co-expression of OCT-4 was examined in VASA-positive cells in the day 14 EBs. A previous report showed that VASA-positive cells co-expressed OCT-4 in monkey EBs and suggested that these cells may correspond to early gonocytes at the post-migration stage [7]. Not only VASA-positive, OCT-4-positive cells were observed, but also VASA-positive, OCT-4-negative cells and VASA-negative, OCT-4-positive cells in EBs (Figure 3I–3N). A small number of VASA-positive cells co-expressed OCT-4 in the EBs examined.

SSEA1 is a possible marker to detect ES cell-derived germ cells in monkeys, since its expression is found not only in primordial germ cells *in vivo* [55] but also in ES cell-derived germ cells *in vitro* in mice and humans [3], [56]. In addition, SSEA1 expression is undetectable in primate ES cells including monkeys and humans unlike mouse ES cells expressing SSEA1 [3], making it easier to distinguish between putative germ cells and ES cells in primates. However, there is little information regarding its expression patterns in germ cells, including ES cell-derived germ cells, in monkeys. The presence of SSEA1 expressing cells in EBs was determined by immunostaining. SSEA1-positive cells were detectable in peripheral region of the EBs, similar to VASA-positive cells, and some of these cells expressed OCT-4 protein (Figure 3C–H).

### Effect of testicular and ovarian cell-conditioned media on germ cell differentiation in monkeys

**Figure 4:**
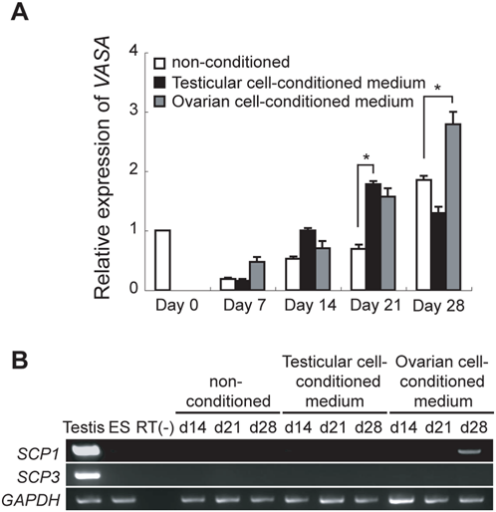
The effect of mouse testicular and ovarian cell-conditioned media on germ cell differentiation in monkey EBs. (A) The *VASA* expression in ES cells (day 0) and EBs (days 7, 14, 21, and 28) in non-conditioned medium, testicular, or ovarian cell-conditioned medium was compared using quantitative RT-PCR. The expression levels of *VASA* in treated EBs are shown relative to *VASA* expression in ES cells (day 0). The data represent the means±standard deviation from triplicate PCR assays. (B) Expression of *SCP1* and *SCP3* in monkey testis (5 years old), ES cells (ES), and developing EBs (days 14, 21, and 28) in non-conditioned medium, testicular, or ovarian cell-conditioned medium was examined using RT-PCR. *GAPDH* was used as an internal control. Statistical significance was tested by Student's *t*-test. Asterisk, *P*<0.01.

A previous report showed that conditioned medium from mouse testicular cells has the ability to derive follicle-like structures containing oocytes from mouse ES cells [4]. To examine the effects of conditioned medium on germ cell differentiation in monkey ES cells, EBs were cultured in mouse testicular or ovarian cell-conditioned medium, and the expression of *VASA* was analyzed using quantitative RT-PCR. *VASA* expression increased slightly between days 14 and 21 in EBs cultured in testicular cell-conditioned medium (Figure 4A). A similar increase was observed between days 21 and 28 in EBs cultured in ovarian cell-conditioned medium (Figure 4A). *VASA* was detected earlier in cells cultured in testicular cell-conditioned medium than in those cultured in non-conditioned medium or in ovarian cell-conditioned medium, suggesting that the initiation of germ cell differentiation in testicular cell-conditioned medium was faster than that in ovarian cell-conditioned medium. The expression of *SCP1* and *SCP3* was examined in the EBs by RT-PCR (Figure 4B), to examine whether EBs cultured in conditioned medium can undergo meiosis. No expression of *SCP3* was detected in the EBs examined, whereas there was an increase of *SCP1* expression in the day 28 EBs cultured in ovarian cell-conditioned medium. Although no morphological features that suggested the formation of follicle-like structures were observed in EBs as reported in mice [4], these results suggest that the conditioned medium induce *VASA* and *SCP1* expression in monkeys.

### Effect of growth factors, BMP4, RA, and SCF on germ cell differentiation in monkeys

**Figure 5:**
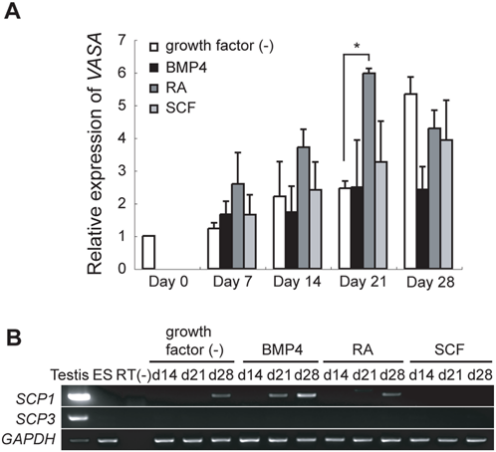
The effect of BMP4, RA and SCF on germ cell differentiation in monkey EBs. (A) *VASA* expression in EBs cultured in the presence or absence of BMP4 (100 ng/ml), RA (1 µM), and SCF (100 ng/ml) was compared using quantitative RT-PCR. The expression levels of *VASA* in treated EBs are shown relative to *VASA* expression in ES cells (day 0). The data represent the means±standard deviation from triplicate PCR assays. (B) The expression of *SCP1* and *SCP3* in monkey testis (5 years old), ES cells (ES), and developing EBs (days 14, 21, and 28) in the presence or absence of BMP4 (100 ng/ml), RA (1 µM), and SCF (100 ng/ml) was examined using RT-PCR. *GAPDH* was used as an internal control. Statistical significance was tested by Student's *t*-test. Asterisk, *P*<0.01.

Previous studies demonstrated the VASA expression to increase with germ cell differentiation from mouse and human ES cells by co-culturing with BMP4-producing cells [57] and the addition of recombinant human BMP4 [6], respectively. The addition of RA and SCF is reported to stimulate the proliferation of primordial germ cells *in vitro* in mice [58], [59]. The addition of RA is also shown to stimulate the meiotic initiation of germ cells *in vitro* in mice [60]. Monkey EBs were cultured in the presence of these factors and the expression of *VASA* was determined using quantitative RT-PCR (Figure 5A). *VASA* expression in EBs cultured with RA was slightly higher than that in EBs cultured in the absence of factors, whereas, no significant increase of *VASA* expression was detected in EBs cultured with BMP4 or SCF in comparison to the EBs in the absence of factors. Next, the expression of *SCP1* and *SCP3* was examined in these EBs. No expression of *SCP3* was detected in the any of the conditioned EBs examined, whereas the expression of *SCP1* was detected from day 21 EBs cultured with BMP4, and detected in day 28 EBs cultured in the absence of growth factors and with RA. The expression of *SCP1* in the EBs cultured with BMP4 was detected earlier than that in EBs cultured in the absence of factors and with RA. These results suggest that these factors can induce some differentiation into a germ cell lineage from monkey ES cells *in vitro*.

## Discussion

Among the early germ cell markers examined, *VASA* is a candidate gene for detecting pre-meiotic germ cell differentiation from monkey ES cells, because its expression is detected earlier in the primordial stage of germ cell development in comparison to that of *PIWI family genes in vivo* in mice and humans [11], [36]–[38], [49]. Because other germ cell marker genes such as *PRDM1, PRMT5, DPPA3*, *IFITM3*, *GDF3*, *c-KIT, CXCR4,* and *NANOS1-3* were expressed in somatic tissues, it is difficult to distinguish putative germ cells from other cells by using only one of these markers. A combination of several germ cell markers will thus be necessary to detect germ cell differentiation from ES cells. *PRDM14* and *DAZL* could be among the candidate genes for detecting germ cells differentiated from ES cells, but both genes were expressed in ES cells to a greater degree than *VASA*. We therefore used *VASA* for the detection of germ cell differentiation from ES cells. VASA was expressed in the testis and was detected from spermatogonia to early spermatids, similar to its expression pattern in mice and humans. *VASA* is thus considered to be a valuable marker for the detection of germ cells in monkeys, as well as in mice and humans.

The *VASA* expression in EBs increased after day 14 in the current culture conditions. In a previous monkey study, the *VASA* expression was reported to increase until day 8 in the EBs [7]. An immunohistological analysis revealed that VASA-positive cells formed clusters at the periphery of the EBs as previously reported in monkeys and humans [5], [7]. This result suggests that the peripheral region of EBs is a suitable microenvironment for germ cell differentiation. This might due to the supply of factors from cells located at the EB surface and/or easier access to factors located in the culture medium. Germ cell differentiation in EBs was further confirmed by immunostaining for SSEA1, OCT-4 and VASA. During mouse development, SSEA1 expression is first detected in primordial germ cells before they migrate into the gonadal ridge, and continues until a post-migratory stage in males and is down-regulated after the onset of meiosis in females [55], [61], [62]. No SSEA1 expressing germ cells were observed in the postnatal testes of 3-year-old and 5-year-old monkeys (data not shown). OCT-4 is found in preimplantation-stage embryos and later confined to primordial germ cells, and then, the expression during male and female gametogenesis exhibits sex-specific down-regulation similar to SSEA1 [63]. The VASA expression is first identified in migrating germ cells through to the post-meiotic stage of male and female gametogenesis in mice and humans [11], [49]. Taken together, at least two types of germ cells were produced in EBs: (i) SSEA1-positive, OCT-4-negative cells, SSEA1-positive, OCT-4-positive cells, and VASA-negative, OCT-4-positive cells represent pre-migratory or migratory germ cells *in vivo,* (ii) VASA-positive, OCT-4-positive cells and VASA-positive, OCT-4 negative cells correspond to early post-migratory germ cells and late post-migratory germ cells, *in vivo*, respectively. These results may suggest that a mixed population of ES cells differentiating into germ cells is present in monkey EBs. Interestingly, a previous study showed that SSEA1 expressing germ cells derived from mouse ES cells express *OCT-4* [3]. SSEA1-positive and OCT-4-positive cells are detectable in fetal gonads in humans [64], [65]. On the other hand, ES cells and cells differentiating into endoderm and mesoderm lineages from ES cells express OCT-4 in mice and humans [66], and SSEA1 expression is found in other type of cells including neural stem cells and mesenchymal stem cells [67], [68]. Further investigation is therefore necessary to characterize the cell populations such as SSEA1-positive, OCT-4-negative cells and VASA-negative, OCT-4 positive cells observed in this study. However, SSEA1 can be a useful cell surface marker for the detection and enrichment of putative germ cells derived from ES cells as reported previously [56]. By using germ cell markers such as SSEA1, OCT-4, and VASA, the identification and characterization of germ cell differentiation from ES cells is possible *in vitro* as well as *in vivo*.

Since *TEKT1* is expressed in spermatocytes and round spermatids in mice [69], the onset of *TEKT1* expression is later than that of *VASA* expression *in vivo*. In the current study, the expression of the two genes was seen in EBs at the same time, thus indicating that the expression patterns of the two genes *in vitro* are different from those *in vivo*. This may reflect the mixed population of germ cells in the EBs, which was demonstrated by the immunostaining analysis. Similar expression patterns of *VASA* and *TEKT1* have been reported in the germ cells differentiated from human ES cells [5]. On the other hand, the current result raises the question why the expression of *SCP1* and *SCP3* could not be detected before the up-regulation of *TEKT1* expression in the EBs. An analysis using knockout mice demonstrated that SCP1-null germ cells survive until around the pachytene stage of meiosis, and interestingly, a small proportion of those cells is able to progress to the diplotene stage [70], and SCP3-null germ cells can develop until around the zygotene stage [71]. *TEKT1* expression in the meiotic stages is detected from around the pachytene stage in mice [69]. Therefore, some germ cells derived from monkey ES cells developed through to a stage correspond to pachytene stage *in vivo*, in which *TEKT1* expression is first detected, even though *SCP1* and *SCP3* are not expressed. However, the lack of any *SCP1* and *SCP3* expression in EBs suggests that the ES cell-derived germ cells did not develop normally through the meiotic stages.

The effects of extrinsic factors was examined using gonadal conditioned media or several growth factors for germ cell differentiation *in vitro* in monkeys. In the both culture conditions, *SCP3* expression was not detected unlike *SCP1*. SCP1 and SCP3 are components of a synaptonemal complex and are expressed in the germ cells from the zygotene and leptotene stages respectively, during meiosis [41], [42], [46]. The localizations and functions in the complex are different between SCP1 and SCP3, and both genes are required for normal meiotic developmental process in mice and humans. On the other hand, the SCP1 expression is detected in germ cells of SCP3 KO mice, though the structure of the synaptonemal complex and localization of SCP1 in the complex are abnormal in comparison to the wild-type germ cells [50]. The lack of any *SCP3* expression in the current experiments might suggest that the germ cell differentiation from ES cells did not go through the meiosis via a completely normal process.

It is unclear whether components of the culture medium other than extrinsic factors affect germ cell differentiation *in vitro* in monkeys. The observation that *SCP1* was expressed in EBs even without extrinsic factors (Figure 5B) may suggest that the components of the culture medium are also one of important factors to effectively induce germ cell differentiation, especially meiotic progression *in vitro*. However, the growth factors that regulate meiosis followed by folliculogenesis and spermatogenesis are still largely unknown in primates. The current findings could provide important clues to determine the culture conditions for promoting the differentiation of primate ES cells into mature gametes, and to understand molecular mechanisms of primate gametogenesis including the timing of germ cell induction, the regulation of germ cell gene expression, and the response to growth factors for germ cell differentiation.

In conclusion, *VASA* is considered to be a valuable marker for detecting germ cells in monkeys, as in mice and humans. *VASA* expression increased during the differentiation of monkey ES cells, indicating that these cells have the ability to differentiate into a germ cell lineage *in vitro*. Research into germ cell development using primate embryos is hampered because of the supply restrictions; however, the use of primate ES cells is a valuable alternative for such studies. Although further research will be required to generate mature gametes in primates, ES cell-derived germ cells may help to clarify the germ cell developmental process *in vitro*.

## References

[1] Hubner K, Fuhrmann G, Christenson LK, Kehler J, Reinbold R (2003). Derivation of oocytes from mouse embryonic stem cells.. Science.

[2] Toyooka Y, Tsunekawa N, Akasu R, Noce T (2003). Embryonic stem cells can form germ cells in vitro.. Proc Natl Acad Sci U S A.

[3] Geijsen N, Horoschak M, Kim K, Gribnau J, Eggan K (2004). Derivation of embryonic germ cells and male gametes from embryonic stem cells.. Nature.

[4] Lacham-Kaplan O, Chy H, Trounson A (2006). Testicular cell conditioned medium supports differentiation of embryonic stem cells into ovarian structures containing oocytes.. Stem Cells.

[5] Clark AT, Bodnar MS, Fox M, Rodriquez RT, Abeyta MJ (2004). Spontaneous differentiation of germ cells from human embryonic stem cells in vitro.. Hum Mol Genet.

[6] Kee K, Gonsalves JM, Clark AT, Pera RA (2006). Bone morphogenetic proteins induce germ cell differentiation from human embryonic stem cells.. Stem Cells Dev.

[7] Teramura T, Takehara T, Kawata N, Fujinami N, Mitani T (2007). Primate embryonic stem cells proceed to early gametogenesis in vitro.. Cloning Stem Cells.

[8] Lasko PF, Ashburner M (1988). The product of the Drosophila gene vasa is very similar to eukaryotic initiation factor-4A.. Nature.

[9] Hay B, Jan LY, Jan YN (1988). A protein component of Drosophila polar granules is encoded by vasa and has extensive sequence similarity to ATP-dependent helicases.. Cell.

[10] Fujiwara Y, Komiya T, Kawabata H, Sato M, Fujimoto H (1994). Isolation of a DEAD-family protein gene that encodes a murine homolog of Drosophila vasa and its specific expression in germ cell lineage.. Proc Natl Acad Sci U S A.

[11] Castrillon DH, Quade BJ, Wang TY, Quigley C, Crum CP (2000). The human VASA gene is specifically expressed in the germ cell lineage.. Proc Natl Acad Sci U S A.

[12] Clark AT, Rodriguez RT, Bodnar MS, Abeyta MJ, Cedars MI (2004). Human STELLAR, NANOG, and GDF3 Genes Are Expressed in Pluripotent Cells and Map to Chromosome 12p13, a Hotspot for Teratocarcinoma.. Stem Cells.

[13] Toyooka Y, Tsunekawa N, Takahashi Y, Matsui Y, Satoh M (2000). Expression and intracellular localization of mouse Vasa-homologue protein during germ cell development.. Mech Dev.

[14] Suemori H, Tada T, Torii R, Hosoi Y, Kobayashi K (2001). Establishment of embryonic stem cell lines from cynomolgus monkey blastocysts produced by IVF or ICSI.. Dev Dyn.

[15] Ponchel F, Toomes C, Bransfield K, Leong FT, Douglas SH (2003). Real-time PCR based on SYBR-Green I fluorescence: an alternative to the TaqMan assay for a relative quantification of gene rearrangements, gene amplifications and micro gene deletions.. BMC Biotechnol.

[16] Ohinata Y, Payer B, O'Carroll D, Ancelin K, Ono Y (2005). Blimp1 is a critical determinant of the germ cell lineage in mice.. Nature.

[17] Yamaji M, Seki Y, Kurimoto K, Yabuta Y, Yuasa M (2008). Critical function of Prdm14 for the establishment of the germ cell lineage in mice.. Nat Genet.

[18] Ancelin K, Lange UC, Hajkova P, Schneider R, Bannister AJ (2006). Blimp1 associates with Prmt5 and directs histone arginine methylation in mouse germ cells.. Nat Cell Biol.

[19] Sato M, Kimura T, Kurokawa K, Fujita Y, Abe K (2002). Identification of PGC7, a new gene expressed specifically in preimplantation embryos and germ cells.. Mech Dev.

[20] Saitou M, Barton SC, Surani MA (2002). A molecular programme for the specification of germ cell fate in mice.. Nature.

[21] Bowles J, Teasdale RP, James K, Koopman P (2003). Dppa3 is a marker of pluripotency and has a human homologue that is expressed in germ cell tumours.. Cytogenet Genome Res.

[22] Lange UC, Saitou M, Western PS, Barton SC, Surani MA (2003). The Fragilis interferon-inducible gene family of transmembrane proteins is associated with germ cell specification in mice.. BMC Dev Biol.

[23] McPherron AC, Lee SJ (1993). GDF-3 and GDF-9: two new members of the transforming growth factor-beta superfamily containing a novel pattern of cysteines.. J Biol Chem.

[24] Caricasole AA, van Schaik RH, Zeinstra LM, Wierikx CD, van Gurp RJ (1998). Human growth-differentiation factor 3 (hGDF3): developmental regulation in human teratocarcinoma cell lines and expression in primary testicular germ cell tumours.. Oncogene.

[25] Ohta H, Yomogida K, Dohmae K, Nishimune Y (2000). Regulation of proliferation and differentiation in spermatogonial stem cells: the role of c-kit and its ligand SCF.. Development.

[26] Cairns LA, Moroni E, Levantini E, Giorgetti A, Klinger FG (2003). Kit regulatory elements required for expression in developing hematopoietic and germ cell lineages.. Blood.

[27] Molyneaux KA, Zinszner H, Kunwar PS, Schaible K, Stebler J (2003). The chemokine SDF1/CXCL12 and its receptor CXCR4 regulate mouse germ cell migration and survival.. Development.

[28] Bucay N, Yebra M, Cirulli V, Afrikanova I, Kaido T (2009). A Novel Approach for the Derivation of Putative Primordial Germ Cells and Sertoli Cells from Human Embryonic Stem Cells.. Stem Cell.

[29] Jaruzelska J, Kotecki M, Kusz K, Spik A, Firpo M (2003). Conservation of a Pumilio-Nanos complex from Drosophila germ plasm to human germ cells.. Dev Genes Evol.

[30] Tsuda M, Sasaoka Y, Kiso M, Abe K, Haraguchi S (2003). Conserved role of nanos proteins in germ cell development.. Science.

[31] Suzuki A, Tsuda M, Saga Y (2007). Functional redundancy among Nanos proteins and a distinct role of Nanos2 during male germ cell development.. Development.

[32] Reijo R, Lee TY, Salo P, Alagappan R, Brown LG (1995). Diverse spermatogenic defects in humans caused by Y chromosome deletions encompassing a novel RNA-binding protein gene.. Nat Genet.

[33] Saxena R, Brown LG, Hawkins T, Alagappan RK, Skaletsky H (1996). The DAZ gene cluster on the human Y chromosome arose from an autosomal gene that was transposed, repeatedly amplified and pruned.. Nat Genet.

[34] Cooke HJ, Lee M, Kerr S, Ruggiu M (1996). A murine homologue of the human DAZ gene is autosomal and expressed only in male and female gonads.. Hum Mol Genet.

[35] Reijo R, Seligman J, Dinulos MB, Jaffe T, Brown LG (1996). Mouse autosomal homolog of DAZ, a candidate male sterility gene in humans, is expressed in male germ cells before and after puberty.. Genomics.

[36] Kuramochi-Miyagawa S, Kimuraa T, Yomogida K, Kuroiwa A, Tadokoro Y (2001). Two mouse piwi-related genes: miwi and mili.. Mech Dev.

[37] Qiao D, Zeeman AM, Deng W, Looijenga LH, Lin H (2002). Molecular characterization of hiwi, a human member of the piwi gene family whose overexpression is correlated to seminomas.. Oncogene.

[38] Lee JH, Schutte D, Wulf G, Fuzesi L, Radzun HJ (2005). Stem-cell protein Piwil2 is widely expressed in tumors and inhibits apoptosis through activation of Stat3/Bcl-XL pathway.. Hum Mol Genet.

[39] Baker SM, Plug AW, Prolla TA, Bronner CE, Harris AC (1996). Involvement of mouse Mlh1 in DNA mismatch repair and meiotic crossing over.. Nat Genet.

[40] Bronner CE, Baker SM, Morrison PT, Warren G, Smith LG (1994). Mutation in the DNA mismatch repair gene homologue hMLH1 is associated with hereditary non-polyposis colon cancer.. Nature.

[41] Meuwissen RL, Offenberg HH, Dietrich AJ, Riesewijk A, van Iersel M (1992). A coiled-coil related protein specific for synapsed regions of meiotic prophase chromosomes.. EMBO J.

[42] Dobson MJ, Pearlman RE, Karaiskakis A, Spyropoulos B, Moens PB (1994). Synaptonemal complex proteins: occurrence, epitope mapping and chromosome disjunction.. J Cell Sci.

[43] Sage J, Martin L, Cuzin F, Rassoulzadegan M (1995). cDNA sequence of the murine synaptonemal complex protein 1 (SCP1).. Biochim Biophys Acta.

[44] Meuwissen RLJ, Meerts I, Hoovers JMN, Leschot NJ, Heyting C (1997). Human synaptonemal complex proteins 1 (SCP1): isolation and characterization of the cDNA and chromosomal localization of the gene.. Genomics.

[45] Heyting C, Dettmers RJ, Dietrich AJ, Redeker EJ, Vink AC (1988). Two major components of synaptonemal complexes are specific for meiotic prophase nuclei.. Chromosoma.

[46] Lammers JH, Offenberg HH, van Aalderen M, Vink AC, Dietrich AJ (1994). The gene encoding a major component of the lateral elements of synaptonemal complexes of the rat is related to X-linked lymphocyte-regulated genes.. Mol Cell Biol.

[47] Reijo RA, Dorfman DM, Slee R, Renshaw AA, Loughlin KR (2000). DAZ family proteins exist throughout male germ cell development and transitfrom nucleus to cytoplasm at meiosis in humans and mice.. Biol Reprod.

[48] Xu EY, Moore FL, Pera RA (2001). A gene family required for human germ cell development evolved from an ancient meiotic gene conserved in metazoans.. Proc Natl Acad Sci U S A.

[49] Noce T, Okamoto-Ito S, Tsunekawa N (2001). Vasa homolog genes in mammalian germ cell development.. Cell Struct Funct.

[50] Costa Y, Speed R, Ollinger R, Alsheimer M, Semple CA (2005). Two novel proteins recruited by synaptonemal complex protein 1 (SYCP1) are at the centre of meiosis.. J Cell Sci.

[51] Stoop H, van Gurp R, de Krijger R, Geurts van Kessel A, Koberle B (2001). Reactivity of germ cell maturation stage-specific markers in spermatocytic seminoma: diagnostic and etiological implications.. Lab Invest.

[52] Tsuneyoshi N, Sumi T, Onda H, Nojima H, Nakatsuji N (2008). PRDM14 suppresses expression of differentiation marker genes in human embryonic stem cells.. Biochem Biophys Res Commun.

[53] Xu M, Zhou Z, Cheng C, Zhao W, Tang R (2001). Cloning and characterization of a novel human TEKTIN1 gene.. Int J Biochem Cell Biol.

[54] Dong J, Albertini DF, Nishimori K, Kumar TR, Lu N (1996). Growth differentiation factor-9 is required during early ovarian folliculogenesis.. Nature.

[55] Fox N, Damjanov I, Martinez-Hernandez A, Knowles BB, Solter D (1981). Immunohistochemical localization of the early embryonic antigen (SSEA-1) in postimplantation mouse embryos and fetal and adult tissues.. Dev Biol.

[56] Tilgner K, Atkinson SP, Golebiewska A, Stojkovic M, Lako M (2008). Isolation of primordial germ cells from differentiating human embryonic stem cells.. Stem Cells.

[57] Ying Y, Qi X, Zhao GQ (2001). Induction of primordial germ cells from murine epiblasts by synergistic action of BMP4 and BMP8B signaling pathways.. Proc Natl Acad Sci U S A.

[58] Koshimizu U, Watanabe M, Nakatsuji N (1995). Retinoic acid is a potent growth activator of mouse primordial germ cells in vitro.. Dev Biol.

[59] Matsui Y, Toksoz D, Nishikawa S, Nishikawa S, Williams D (1991). Effect of Steel factor and leukaemia inhibitory factor on murine primordial germ cells in culture.. Nature.

[60] Bowles J, Knight D, Smith C, Wilhelm D, Richman J (2006). Retinoid signaling determines germ cell fate in mice.. Science.

[61] Lacham-Kaplan O (2004). In vivo and in vitro differentiation of male germ cells in the mouse.. Reproduction.

[62] Chuma S, Nakatsuji N (2001). Autonomous transition into meiosis of mouse fetal germ cells in vitro and its inhibition by gp130-mediated signaling.. Dev Biol.

[63] Pesce M, Gross MK, Scholer HR (1998). In line with our ancestors: Oct-4 and the mammalian germ.. Bioessays.

[64] Kerr CL, Hill CM, Blumenthal PD, Gearhart JD (2008). Expression of pluripotent stem cell markers in the human fetal testis.. Stem Cells.

[65] Kerr CL, Hill CM, Blumenthal PD, Gearhart JD (2008). Expression of pluripotent stem cell markers in the human fetal ovary.. Hum Reprod.

[66] Mossman AK, Sourris K, Ng E, Stanley Eg, Elefanty AG (2005). Mixl1 and oct4 proteins are transiently co-expressed in differentiating mouse and human embryonic stem cells.. Stem Cells Dev.

[67] Capela A, Temple S (2006). LeX is expressed by principle progenitor cells in the embryonic nervous system, is secreted into their environment and binds Wnt-1.. Dev Biol.

[68] Polisetty N, Fatima A, Madhira SL, Sangwan VS, Vemuganti GK (2008). Mesenchymal cells from limbal stroma of human eye.. Mol Vis.

[69] Larsson M, Norrander J, Gräslund S, Brundell E, Linck R (2000). The spatial and temporal expression of Tekt1, a mouse tektin C homologue, during spermatogenesis suggest that it is involved in the development of the sperm tail basal body and axoneme.. Eur J Cell Biol.

[70] de Vries FA, de Boer E, van den Bosch M, Baarends WM, Ooms M (2005). Mouse Sycp1 functions in synaptonemal complex assembly, meiotic recombination, and XY body formation.. Genes Dev.

[71] Yuan L, Liu JG, Hoja MR, Wilbertz J, Nordqvist K (2002). Female germ cell aneuploidy and embryo death in mice lacking the meiosis-specific protein SCP3.. Science.

